# Profound Iron Deficiency Anemia Associated With Endoscopically Visualized Nematode Infestation Mimicking Gastric Malignancy: A Case Report From Trinidad and Tobago

**DOI:** 10.7759/cureus.111538

**Published:** 2026-06-26

**Authors:** Aamir Ali, Tarun V Ramlogan

**Affiliations:** 1 Internal Medicine, Sangre Grande Hospital, Sangre Grande, TTO

**Keywords:** gastric wall thickening, hookworm infestation, iron deficiency anemia, nematode infection, soil-transmitted helminths

## Abstract

Severe iron deficiency anemia (IDA) secondary to parasitic infestation is uncommon in modern clinical practice but remains an important diagnostic consideration in endemic regions and vulnerable populations. We present the case of a 61-year-old male patient who presented to the emergency department following a syncopal episode and fall and was found to have profound anemia with an initial hemoglobin level of 2.5 g/dL and a serum ferritin of 1.96 ng/ml. Initial diagnostic workup with contrast-enhanced computed tomography of the abdomen and pelvis excluded acute intra-abdominal hemorrhage but demonstrated marked thickening of the gastric fundus and body, raising concern for an underlying gastric malignancy. Laboratory investigations additionally revealed raised eosinophil percentage on complete blood count analysis. Following hemodynamic stabilization and packed red blood cell transfusion, the patient underwent esophagogastroduodenoscopy. Endoscopic evaluation revealed short-segment Barrett’s esophagus (Prague classification C0M2) and multiple motile nematodes attached to the duodenal mucosa, consistent with an endoscopically visualized nematode infestation involving the proximal small bowel. In the context of endemic exposure, raised eosinophil percentage, profound microcytic anemia, and low ferritin, this was considered morphologically suggestive of hookworm infestation. Further dietary and social history revealed habitual barefoot outdoor exposure and regular consumption of wild meat, supporting environmental risk factors for parasitic infection. Stool microscopy was negative, and histopathology did not identify parasitic organisms or malignancy. The patient was treated with albendazole and proton pump inhibitor therapy, with subsequent clinical improvement, recovery of hemoglobin levels following transfusion and anti-helminthic treatment, and sustained hematologic stability on follow-up. This case highlights the importance of considering parasitic infection in the differential diagnosis of severe microcytic anemia and gastric wall thickening, particularly in endemic settings. It also demonstrates how infectious gastrointestinal pathology may mimic advanced malignancy on cross-sectional imaging.

## Introduction

Iron deficiency anemia (IDA) remains the most prevalent form of anemia worldwide and continues to represent a significant public health burden, particularly in low-resource environments and tropical regions. In older adults, important causes of severe anemia include occult gastrointestinal blood loss, gastrointestinal malignancy, nutritional deficiency, malabsorption, chronic inflammatory disease, hemolysis, bone marrow disorders, and other hematological conditions. However, chronic parasitic infection remains an important yet frequently under-recognized cause of IDA in endemic settings. Among helminthic infections, hookworm infection, primarily caused by *Necator americanus* in the Caribbean and *Ancylostoma duodenale* in other subtropical regions, represents a well-recognized mechanism of profound and progressive iron depletion [1-4]. Transmission occurs through percutaneous penetration by infective larvae in contaminated soil and is therefore associated with agricultural exposure and habitual barefoot walking in endemic communities [2-4].

Once inside the host, these hematophagous nematodes migrate to the upper gastrointestinal tract, using specialized mouthparts to securely anchor themselves into the mucosa of the duodenum and proximal jejunum. This mechanical attachment allows the mature worms to actively ingest host blood and excrete anticoagulants, precipitating chronic, high-volume occult blood loss [5-7]. While a significant proportion of infected individuals exhibit mild constitutional symptoms or remain entirely asymptomatic, an aggressive parasite burden can exhaust systemic iron stores, culminating in life-threatening microcytic anemia, severe hypoalbuminemia, and high-output cardiac failure or syncope [1,5,6].

The traditional diagnostic gold standard relies on the identification of helminth ova via stool microscopy. However, the diagnostic sensitivity of fecal standard testing is frequently compromised by low parasite counts, intermittent egg-shedding cycles, or pre-analytical specimen handling errors [1,4]. Consequently, the direct endoscopic visualization of active, motile helminths within the proximal small bowel during esophagogastroduodenoscopy (EGD) has emerged as an invaluable diagnostic modality, turning an incidental finding into a definitive, life-saving therapeutic pivot [5-8].

Although hookworms typically colonize the proximal small intestine, rare gastric mucosal involvement has been reported and may produce inflammatory changes resembling early gastric carcinoma [9]. We present the unique case of a 61-year-old male patient from rural Trinidad who presented acutely following a syncopal episode, found to have a near-fatal index hemoglobin of 2.5 g/dL. The initial contrast-enhanced computed tomography (CT) demonstrated marked gastric wall thickening, while endoscopy concurrently identified multiple nematodes attached to the duodenal mucosa, morphologically suggestive of hookworm infestation in the clinical and epidemiological context.. This case, therefore, illustrates an important diagnostic pitfall in the evaluation of suspected gastric malignancy and highlights the necessity of maintaining a high index of clinical suspicion for parasitic etiologies in tropical zones, demonstrating that fully reversible infectious infestations can convincingly mimic advanced gastrointestinal cancers on modern cross-sectional imaging.

## Case presentation

A 61-year-old man presented to the emergency department with a two-day history of generalized weakness and several days of dizziness. He had experienced a sudden syncopal episode with a fall on the day before presentation. He also described minor, previously uninvestigated suprapubic discomfort. There was no documented history of melena, hematemesis, hematochezia, weight loss, or altered bowel habits. He had no known previous diagnosis of anemia or gastrointestinal malignancy. On examination, he had marked conjunctival and palmar pallor but remained hemodynamically stable, with a blood pressure of 99/52 mmHg and a pulse rate of 55 beats per minute.

Initial laboratory investigations revealed severe anemia with a hemoglobin level of 2.5 g/dL and a microcytic red cell indices pattern. Serum ferritin was markedly reduced at 1.96 ng/mL, supporting IDA. The initial laboratory investigations are summarized in Table 1.

**Table 1:** Initial laboratory investigations on admission WBC: white blood cell count; MCV: mean corpuscular volume; BUN: blood urea nitrogen; CEA: carcinoembryonic antigen; CA 19-9: carbohydrate antigen 19-9; PSA: prostate-specific antigen

Parameter	Patient Value	Reference range
Hemoglobin (g/dL)	2.5	12.90-15.90
MCV (Fl)	57.99	81.1-96.0
WBC count (×10^3 ^u/L)	4.69	3.70-10.10
Platelet (×10^3 ^u/L)	260	155-366
Eosinophils count	0.43	0.03-0.44
Eosinophil Percentage	9.19	0.6-7.30
Ferritin (ng/ml)	1.96	13.0-400
BUN (mg/dL)	10.0	6.00-20.0
Creatinine (mg/dL)	0.7	0.5-1.20
CEA (ng/ml)	1.57	0.0-4.7
CA 19.9 (U/ml)	9.32	0.0-27.0
PSA (ng/ml)	0.5	0.80-2.0

**Figure 1:**
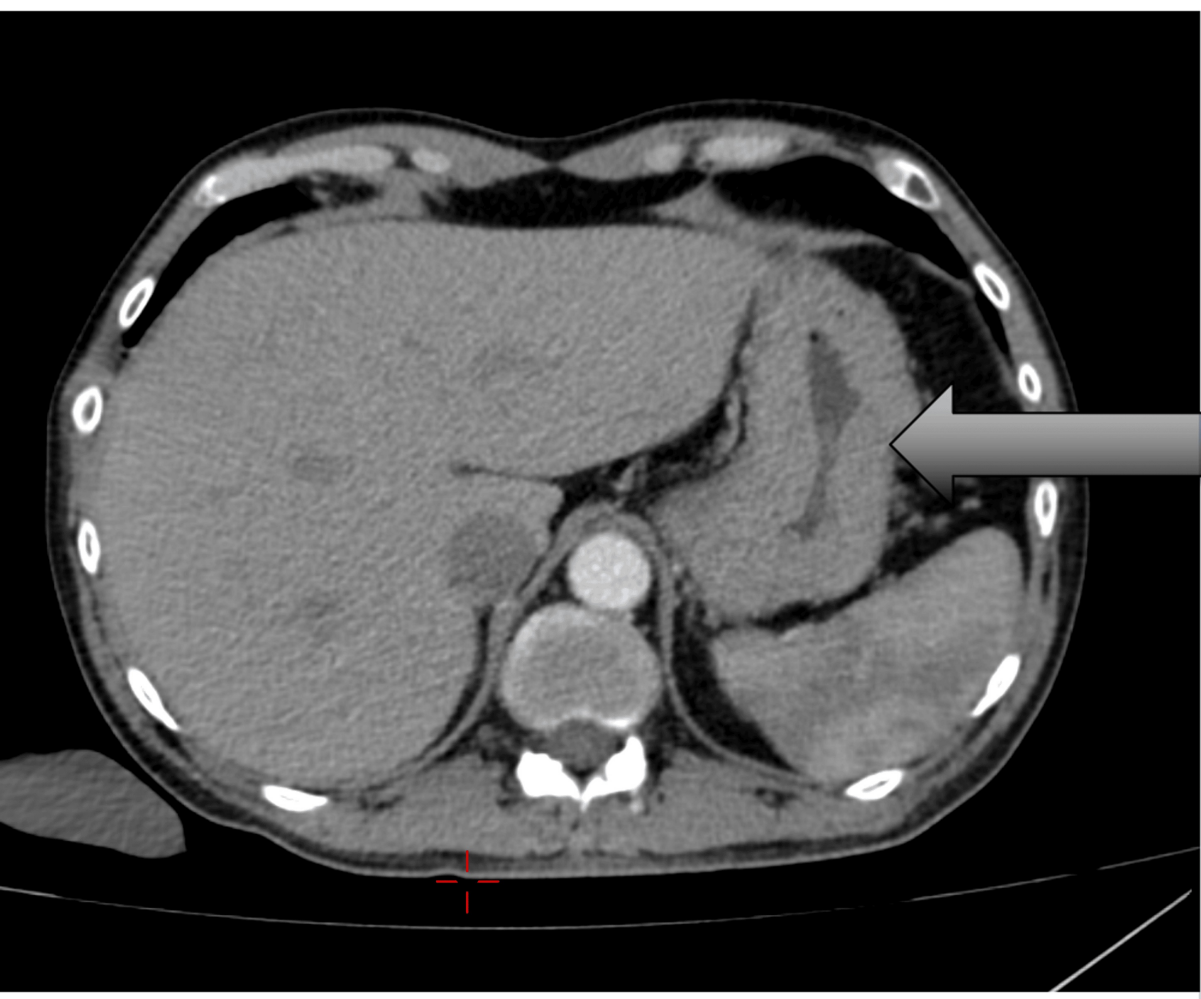
Fundus/body wall thickening seen on CT

Given the severity of anemia in the context of syncope, urgent imaging was performed to exclude traumatic injury and occult intra-abdominal pathology. CT imaging of the head and neck showed no acute intracranial hemorrhage or fractures. Contrast-enhanced CT of the abdomen and pelvis demonstrated marked gastric wall thickening involving the fundus and body, raising concern for possible malignancy, with no evidence of active bleeding or intra-abdominal fluid collection (Figure 1).

The patient was admitted for optimization prior to endoscopic evaluation. A staged transfusion of packed red blood cells was administered, achieving a post-transfusion hemoglobin of 7.45 g/dL, allowing safe endoscopic assessment (Table 2).

**Table 2:** Hemoglobin trend during admission and follow-up

Time point	Hemoglobin level (g/dL)	Reference range
Admission	2.5	12.90-15.90
Admission (repeat)	2.2	12.90-15.90
Admission (post blood transfusion)	3.62	12.90-15.90
Day 1	4.33	12.90-15.90
Day 2 (post blood transfusion)	5.44	12.90-15.90
Day 3	5.67	12.90-15.90
Day 4 (post blood transfusion x 2)	7.45	12.90-15.90
Day 5 (day of discharge)	7.2	12.90-15.90
Four-week follow-up	8.22	12.90-15.90
Four-month follow-up	12.22	12.90-15.90

**Figure 2:**
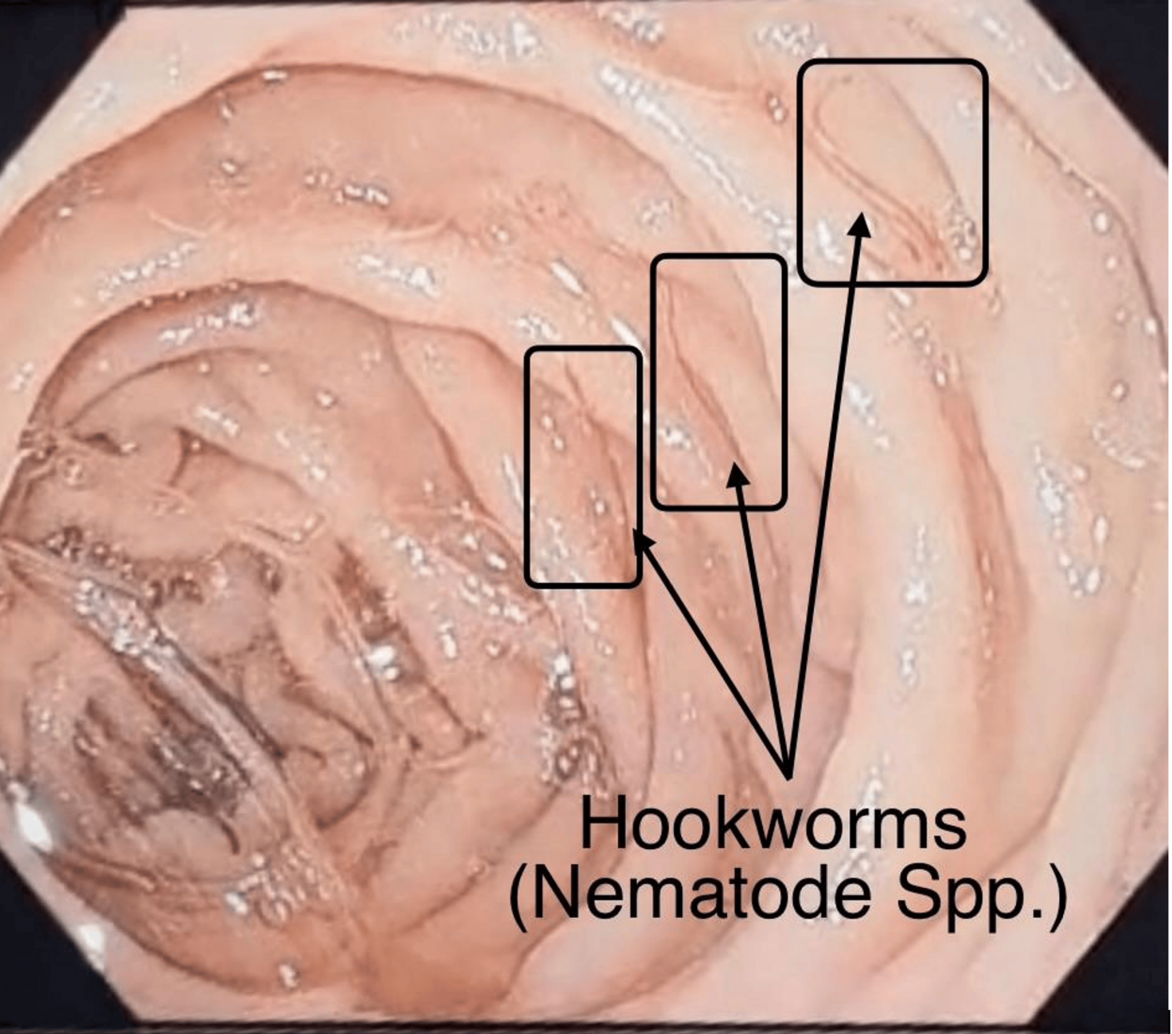
Upper gastrointestinal endoscopy demonstrating multiple slender nematodes attached to the duodenal mucosa, consistent with an endoscopically visualized nematode infestation involving the proximal small bowel. In the clinical and epidemiological context, the appearance was considered suggestive of hookworm infestation.

The following day, the patient remained clinically stable and underwent esophagogastroduodenoscopy without complication. Endoscopy demonstrated short-segment Barrett’s esophagus (Prague classification C0M2). Within the duodenum, multiple motile nematodes were visualized attached to the mucosa, consistent with an endoscopically visualized nematode infestation involving the proximal small bowel (Figure 2). During the procedure, biopsies were obtained from the gastric mucosa corresponding to the radiologically abnormal region, the esophagogastric junction, and the duodenum. These specimens were submitted for histopathological examination to assess for malignancy, mucosal inflammation, and parasitic organisms.

Histopathological examination of the gastric biopsies demonstrated active moderate chronic gastritis without evidence of dysplasia or malignancy. Esophagogastric junction biopsies showed mild chronic gastritis, while duodenal biopsies demonstrated benign duodenal mucosa. No parasitic organisms were identified on histopathological examination. The histopathology findings are summarized in Table 3.

**Table 3:** Histopathology findings from upper gastrointestinal biopsies

Site	Histopathology Findings
Stomach	Active moderate chronic gastritis
Esophagogastric junction	Mild chronic gastritis
Duodenum	Benign duodenal mucosa
Malignancy	Not identified
Parasitic organisms	Not identified

Following endoscopic findings, further history revealed habitual barefoot outdoor exposure and regular consumption of wild meat. In combination with endoscopic appearance and endemic prevalence, a presumptive diagnosis of helminthic infestation was made. Treatment with albendazole 400 mg orally was initiated, with a planned repeat dose in two weeks, alongside proton pump inhibitor therapy with pantoprazole. The patient remained clinically stable throughout admission and was discharged with improving hemoglobin levels and outpatient follow-up arranged.

## Discussion

This case highlights several important clinical lessons. Although IDA in older adults often prompts an urgent search for gastrointestinal malignancy, parasitic infection should remain an important differential diagnosis, particularly in endemic and rural settings. In this patient, the anemia was profound, with an admission hemoglobin of 2.5 g/dL, and contrast-enhanced CT of the abdomen demonstrated marked gastric wall thickening involving the fundus and body, raising concern for malignancy. However, endoscopic evaluation revealed multiple motile nematodes attached to the duodenal mucosa, while histopathology showed no evidence of malignancy. This illustrates how gastrointestinal infection may coexist with radiological findings concerning for malignancy and should remain part of the differential diagnosis in endemic settings.

Hookworms remain one of the most common causes of helminth-associated IDA, particularly in rural settings, with approximately 25% of the world’s population at risk of soil-transmitted helminth infections [2]. These blood-feeding parasites attach to the mucosa of the duodenum and proximal jejunum, where they ingest host blood and release anticoagulant substances, resulting in chronic occult gastrointestinal blood loss. Although many infected individuals remain asymptomatic or experience only mild symptoms, a heavy parasite burden may cause progressive iron depletion and severe microcytic anemia, with complications including hypoalbuminemia, syncope, and high-output cardiac failure.

The Centers for Disease Control and Prevention (CDC) notes that hookworm infection is most prevalent in settings with poor sanitation, including areas where open defecation occurs or infected human feces are used as fertilizer [4]. In keeping with this, our patient had recognized risk factors for transmission, including habitual barefoot outdoor exposure and regular contact with potentially contaminated soil in a rural endemic setting.

An environmental history is therefore an important component of the assessment of patients with suspected parasitic causes of IDA. In this case, the patient’s habitual barefoot outdoor exposure in rural Trinidad provided a plausible route for soil-mediated transmission. Hookworm eggs passed in feces hatch in the soil and develop into infective filariform larvae capable of penetrating intact human skin. Transmission occurs predominantly through contact with contaminated soil, particularly during barefoot exposure, rather than through direct person-to-person spread or contact with fresh feces [3].

This case also highlights the limitations of relying exclusively on stool microscopy or histopathology in the diagnosis of helminthic infection. Only one stool specimen was examined and was negative for ova, cysts, and parasites, while no parasitic organisms were identified on histopathological examination. However, the sensitivity of stool microscopy may be reduced by a low parasite burden, intermittent ova shedding, and suboptimal specimen collection or handling. Therefore, a single negative stool examination does not exclude helminthic infection when the clinical, epidemiological, and endoscopic findings are strongly supportive. In this patient, multiple nematodes were directly visualized within the proximal small bowel and were morphologically suggestive of hookworm infection. When considered alongside their anatomical distribution, habitual barefoot soil exposure, profound IDA, raised eosinophil percentage, and clinical and hematological response to albendazole, these findings supported a presumptive diagnosis of hookworm infection. Nevertheless, definitive species identification was not obtained, and this remains an important limitation of the case.

Iron studies beyond serum ferritin were unavailable at our institution. However, the combination of marked microcytosis and a profoundly reduced ferritin level strongly supported IDA. These findings demonstrate the importance of interpreting laboratory results alongside the clinical, epidemiological, and endoscopic features of the case.

The differential diagnosis for profound iron deficiency anemia associated with gastric wall thickening in an older adult is broad and includes gastric adenocarcinoma, gastric lymphoma, gastrointestinal stromal tumor, severe gastritis, peptic ulcer disease, and other inflammatory or infiltrative disorders [10]. Other potential causes of severe iron deficiency anemia include occult gastrointestinal bleeding from colorectal malignancy or vascular lesions, nutritional deficiency, malabsorption, and chronic blood loss from other sources [11]. In this patient, gastric malignancy was initially a major concern because of the marked thickening involving the gastric fundus and body on CT and the severity of the anemia. However, gastric biopsies demonstrated active moderate chronic gastritis without dysplasia or malignancy, biopsies from the esophagogastric junction showed mild chronic gastritis, and duodenal biopsies demonstrated benign mucosa. No parasitic organisms were identified on histopathological examination. Although multiple nematodes were directly visualized within the duodenum and the patient subsequently demonstrated sustained clinical and hematological recovery following albendazole treatment, a direct causal relationship between the nematode infestation and the gastric wall thickening could not be established. The response to treatment reduced the likelihood of an advanced malignant process, but did not prove that the nematode infestation caused the radiological abnormality.

Management required both urgent stabilization and treatment of the suspected underlying cause. Blood transfusion was necessary because of symptomatic, near-fatal anemia and allowed safe optimization for EGD. The patient was subsequently treated with albendazole and proton pump inhibitor therapy, with marked symptomatic improvement and progressive hematological recovery. His hemoglobin increased to 8.22 g/dL at four weeks and 12.93 g/dL at four months, with normalization of the mean corpuscular volume (MCV) to 83 fL and an increase in ferritin to 200 ng/mL. This case, therefore, illustrates the importance of stabilizing patients with severe anemia while continuing to pursue the underlying diagnosis in a timely manner.

More broadly, this case reflects the continuing public health importance of soil-transmitted helminth infection in tropical settings, where poverty, inadequate sanitation, and environmental exposure influence patterns of disease. The World Health Organization’s 2030 strategy for soil-transmitted helminths focuses on reducing morbidity among at-risk groups, strengthening preventive treatment programs, improving sanitation and hygiene, and integrating soil-transmitted helminth control into primary healthcare [3]. Recognition of individual cases remains important both for patient care and for reinforcing awareness of preventable parasitic disease in endemic regions.

Overall, this case emphasizes the need for a high index of suspicion for parasitic causes of IDA in Trinidad and similar endemic settings. Even in older adults, and when imaging raises concern for malignancy, clinicians should consider potentially reversible infectious causes when the exposure history and endoscopic findings are supportive. Early recognition may prevent diagnostic delay and allow prompt institution of appropriate therapy.

## Conclusions

Profound IDA occurring in the setting of an endoscopically visualized nematode infestation remains clinically relevant in rural tropical practice. In patients presenting with profound microcytic anemia, particularly in the context of rural residence, barefoot soil exposure, and limited sanitation, helminthic infection should remain an important differential diagnosis. This case demonstrates that an endoscopically visualized nematode infestation morphologically suggestive of hookworm infection may coexist with radiological findings suspicious for malignancy. However, because stool microscopy and histopathology were negative and definitive species identification was not obtained, the diagnosis of hookworm infection remained presumptive. This case highlights that a single negative stool examination does not fully exclude helminthic infection when the clinical, epidemiological, and endoscopic findings are strongly supportive.
